# Impact of international travel dynamics on domestic spread of 2019-nCoV in India: origin-based risk assessment in importation of infected travelers

**DOI:** 10.1186/s12992-020-00575-2

**Published:** 2020-05-12

**Authors:** Sachin S. Gunthe, Satya S. Patra

**Affiliations:** 1 EWRE Division, Dept. of Civil Engineering, Indian Institute of Technology Madras, Chennai, 600 036 India; 2 Transportation Engineering Division, Dept. of Civil Engineering, Indian Institute of Technology Madras, Chennai, 600 036 India

**Keywords:** COVID-19, India, Travel risk assessment, Airport screening

## Abstract

The recent pandemic caused by the 2019 outbreak of novel coronavirus (2019-nCoV or COVID-19) has affected more than 3.0 million people resulting ~ 212,000 deaths across 215 countries/territories as on 28th April 2020. The importation of the cases owing to enormous international travels from the affected countries is the foremost reason for local cycle of transmission. For a country like India, the second most populous country in the world with ~ 1.35 billion population, the management and control of 2019-nCoV domestic spread heavily relied on effective screening and strict quarantine of passengers arriving at various international airports in India from affected countries. Here, by extracting the data from FLIRT, an online airline database for more than 800 airlines, and scanning more than 180,000 flights and 39.9 million corresponding passenger seats during 4th – 25th March, we show that India experienced the highest risk index of importing the passengers from middle eastern airports. Contrary to perception, travelers from China imposed lowest risk of importing the infected cases in India. This is clearly evident form the fact that while the number of infected cases were on the peak in China India was one of the least affected countries. The number of cases in India started exhibiting a sharp increase in the infected cases only after the European countries and USA recorded large number of infected cases. We further argue that while the number of cases in middle eastern countries may still be very low, the airports in middle eastern countries, particularly Dubai, being one of the largest transit hubs for international passengers, including arriving in India, might have posed a higher risk of getting infected with 2019-nCoV. We suggest that any future travel related disease infection screening at the airports should critically assess the passengers from major transit hubs in addition to affected country of origin.

## Background

In December 2019, health authorities from Wuhan, Hubei province in China, local hospital were monitoring and reported group of pneumonia cases [1]. The specific pathogen triggering this viral pneumonia, on 7th January 2020, was identified by Chinese Center for Disease Control and Prevention, and was named 2019-nCov, the new coronavirus, by the World Health Organization [2]. Further investigations traced the origin of 2019-nCoV to a local Huanan seafood market. Since then 2019-nCoV was exported to many cities across the globe [3] and ~ 3.09 million people have been infected due to 2019-nCoV resulting in more than 200,000 deaths across the globe as on 28th April 2020, marking this as one of the biggest pandemics in this century. Since the detection of the first case in December 2019 in China, the 2019-nCoV has already reached in more than 200 countries/territories, with ~ 62% of infected cases are being in USA (26.3%), Spain (10.4%), Italy (10.2%), Germany (7.9%), and France (7.1%). There is no doubt that 2019-nCoV outbreak is posing the serous risk for human well-being and the world economy [4].

India, the second most populous country in the world with total population of ~ 1.35 billion inhabitants, registered its first case on 30th of January 2020, as a 20-year-old female medical student, along with two other students, travelled from Wuhan to the south-western state, Kerala. Since then India has registered ~ 657 cases till 25th March, out of which ~ 62% were reported to be the imported cases travelling from various other affected countries. Eventually, India then suspended all international flights to curb the domestic spread due to 2019-nCoV on 25th March 2020. Right from the early days of the pandemic, body temperature screening to verify if the traveler has a fever, as an indication for the infection, as a major test was introduced at all international airports in India. However, it has been noticed that asymptomatic contact transmission of 2019-nCoV and passengers successfully passing the symptom-based screening have been tested positive for 2019-nCoV [5]. If such passengers passing thermal screening test are later tested positive for 2019-nCoV, challenges this approach as body temperature screening could miss the passenger incubating the disease or concealing the fever by other means during the travel [5]. Further to this India has also introduced an additional step of marking the passenger arriving from most affected countries and compelling self-isolation or home quarantine for the 14 days upon arriving in India.

The timing of 2019-nCoV outbreak and aftermath encompassing the lunar new year celebrations in China coincided with very large amount of human movement between China and rest of the world imposing major threat for rapid spread of infection [1]. Owing to such risk it was important that countries take timely decisions to suspend the flights from affected areas, mainly from Chinese cities in this case. Amid the 2019-nCoV outbreak, WHO has observed that many countries are violating the International Health Regulations (IHR) defying the travel restriction against China during 2019-nCoV outbreak [6]. Further, many researchers in this direction have already made the assessments of risk of 2019-nCoV spread from China to the rest of the world including the effect of travel restriction on spread of 2019-nCoV [4, 7, 8]. Thus, while the outbound risk from China to many other countries/territories have been investigated in detail, the inbound risk of 2019-nCoV infection particularly to hugely populated India, however, is not investigated. Here, in a maiden attempt we estimate the risk of importation of 2019-nCoV due to international arrivals in India by screening available data from 19 international airports across the globe and developing the index for risk assessment.

## Main text

### Data collection

In the present study, to calculate the importation risk of 2019-nCoV infection we collected the number of passengers for given airports of an origin country travelling to various international airports in India. We also accounted the total number of 2019-nCoV infected cases in the country of origin. Information about the total number of infected cases in the country of origin was obtained by extensive online search of more than five different sources, which mainly included the government sources and were rigorously verified and validated for any inconsistencies. To obtain the count of air passengers from a origin airport in given country, we relied on a web-based analytical tool called FLIRT [9]. The major application of FLIRT is to predict the air travel network flow between origin and destination airports throughout the world, where user can choose the origin and destination pair. Note that the FLIRT does not take an account of transit passenger is making between origin to destination and would always assign the last boarding airport as an origin, once entered in final destination. This web application allows user perform passenger simulations based on the air traffic between source and destination. To simulate the results, it uses a database of flight schedules of more than 800 airlines [9]. Researchers in the past [1, 9] have used this web interface to estimate the number of infected travelers originating from different countries to the respective destinations. For India, in this study, the countries selected for importation risk index calculation were China, France, Germany, Iran, Italy, Kuwait, Oman, Qatar, Singapore, Spain, Thailand, United Arab Emirates (UAE), United Kingdom (UK), and United States of America (USA), which constituted ~ 70% of the total infected cases. For simplicity in analysis, the countries Iran, Kuwait, Oman, Qatar, and UAE were grouped together as Middle Eastern countries. The major international airports from all the above-mentioned countries were selected to simulate the air traffic flow. The IATA (International Air Transport Association) codes for all the selected airports are listed in Table 1.

**Table 1:** The list of countries and corresponding airports included in the present study to simulate the flight connections and screen the passengers. The airports abbreviations are the IATA (International Air Transport Association) codes. WUH: Wuhan, PEK: Beijing Capital International Airport, CAN: Guangzhou Baiyun International Airport, CDG: Paris-Charles De Gaulle, ORY: Orly Airport, FRA: Frankfurt Airport, MUC: Munich International Airport, FCO: Leonardo da Vinci International Airport, MXP: Malpensa Airport Milan, DXB: Dubai International Airport, SHJ: Sharjah International Airport, AUH: Abu Dhabi International Airport, DOH: Hamad International Airport Doha, KWI: Kuwait International Airport, MCT: Muscat International Airport, IKA: Imam Khomeini International Airport Tehran, MAD: Madrid-Barajas Adolfo Suárez Airport, SIN: Singapore Changi Airport, BKK: Suvarnabhumi Airport Bangkok, HKT: Phuket International Airport, LHR: Heathrow Airport London, JFK: John F. Kennedy International Airport New York, ORD: O’Hare International Airport, Chicago, and SFO: San Francisco International Airport

Country	Airports
China	WUH, PEK, CAN
France	CDG, ORY
Germany	FRA, MUC
Italy	FCO, MXP
Middle Eastern Countries	DXB, SHJ, AUH, DOH, KWI, MCT, IKA
Spain	MAD
Singapore	SIN
Thailand	BKK, HKT
UK	LHR
USA	JFK, ORD, SFO

The FLIRT web application allows two different types of flight data simulation. The first mode includes the simulation of total seats offered by airlines between origin and destination airports, and the other mode simulates the total passengers travelled between airports. The second mode, however, has a significant disadvantage that it allows only 20,000 passengers to be simulated at a time, which is an upper bound on the total number of passengers that can be simulated. Such an upper bound can have a strong bias in the analysis, particularly for this kind of studies. Therefore, the first simulation mode was preferred here. This selection would not affect the analysis, as FLIRT assumes that the number of seats between two airports is directly proportional to the number of passengers between them [9]. Thus, for each airport mentioned in Table 1, the simulation was performed using FLIRT as per the first criteria mentioned above. This simulation was carried out for the period of 4th March 2020 till 24th March 2020. This period was selected because the number of 2019-nCoV cases in India started to grow significantly from 4th of March, and India imposed an international travel ban from 24th of March. During this period India has observed a screening at all international airports across the country. After each simulation, the result was filtered for all the India bound flights for the respect chosen airports, and thus the total number of passenger seats and the number of Indian bound passenger seats were estimated from each airport of the origin. This data along with the number of infected persons in the origin airport country, from the various authenticated sources was then used to estimate an importation risk index.

### Importation risk index calculation

The specific methodology to derive importation risk index for a for a given destination was demonstrated by many researchers [1, 10]. Based on these studies we used the similar methodology to derive the importation risk index using the following equation.

$$ Importation\ risk\ index={\sum}_{i=1}^na\frac{P}{T}\frac{I}{N} $$

where, *a* is the transmission rate, which is assumed to be 2.5 (recommended by WHO), *P* is the total number of India bound seats from the international airports in the origin country, and *T* is the total number of seats in all the airlines originating from the selected airports or origin country. *I* is the total number of 2019-nCoV infected patient in the origin country and *N* is the population of the origin country. The Importation Risk Index is a weighted risk number and is proportional to the risk possessed by a country to import 2019-nCoV cases into India.

The estimation was carried out for 24 airports selected across ten countries, and a Pearson Correlation coefficient test between the estimated risk index and the total number of imported 2019-nCoV cases in India from these countries up to 24th March, as reported by the government, was performed. The detailed results are presented below.

## Results

**Fig. 1:**
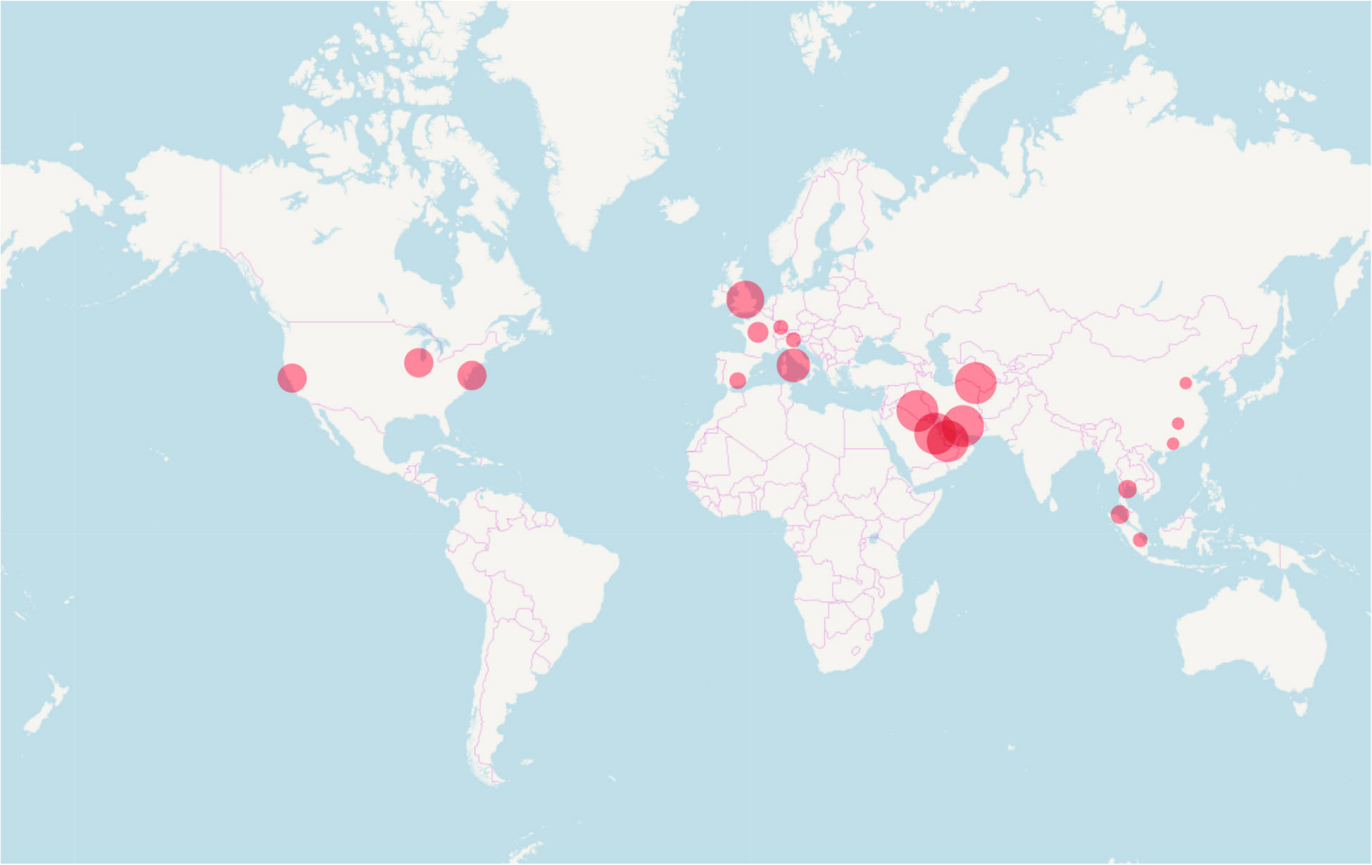
Global map indicating the location of 24 different airports from 10 different countries considered in this study. The size of the circles represents the importation risk index. The political boundaries are for the representation only as provided by the original data source and authors not necessarily agree with the same

**Fig. 2:**
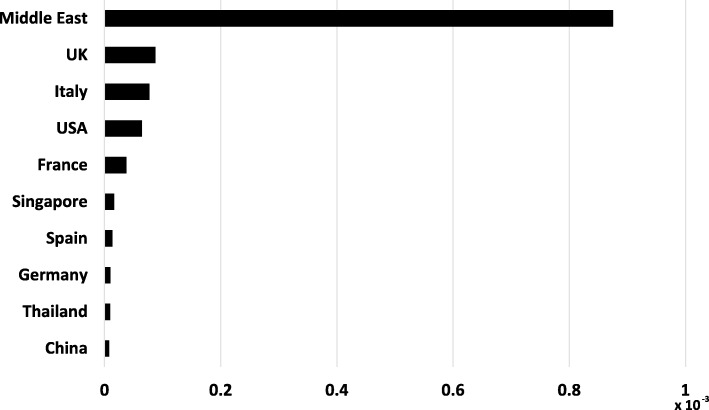
Chart representing the importation risk index for various countries derived between 4th March 2020 and 24th March 2020 by taking an account of 24 international airports in respective countries

**Fig. 3:**
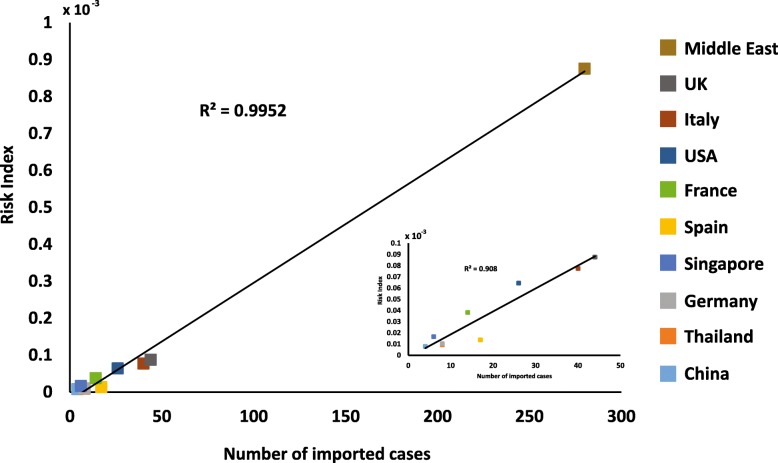
The scatter plot between the actual number of cases in India imported from ten countries (corresponding airports are listed in Tab. 1) and derived importation risk index. The inset figure is similar to the main figure except data point pertaining to middle east is excluded

In this analysis, a total number of 187,755 flights were simulated across 24 airports and ten countries estimating around 33.9 million passenger seats. The importation risk indices of the individual countries for various airports estimated are shown in Fig. 1. As evident from Fig. 1 the Middle Eastern countries posed the highest risk indices followed by UK, Italy, and USA. It is important here to note that the current simulation does not take an account of transit passenger; for example, a passenger originating from JFK arriving in BOM via DXB, would be assigned as a passenger from DXB and not from JFK. The estimated importation risk index is consistent with observed data obtained from various government sources that 7 airports from middle east brought in the maximum number of 2019-nCoV infected passengers (~ 300) in India until the international flights were suspended. It is important to note that Emirates Airlines, a Dubai based airlines, operates 172 flights per week to India serving nine destinations (Emirates and India; partners in economic growth 2018), which is by far highest number of flights and destination by any other foreign airlines. Further, in 2017–18 Emirates has reported 87% of the seat factor and assuming that 50% of them are transit passengers arriving from Europe and USA, thus by far, it is an extremely fair and valid assumption that Dubai airport (and other transit airports) in absence of adequate preventive measures, could be a potential hotspot in future for transmitting infection during COVID-19 like outbreaks. As shown in Fig. 2 the importation risk index for various countries (airports clubbed together) clearly showing the middle east having the highest risk index. A certain amount of risk, although significantly lower than middle east airports, was also posed by passengers originating at LHR and taking the direct flights to India. As expected, the third highest importation risk was posed by Italy, which was one of the worst affected countries during our study window. It is also to be noted that Indian Government has operated special flights to bring back the stranded citizen from Italy. Surprisingly and contrary to expectation the direct travelers from China posed the lowest risk (Fig. 2), this could be due to potentially much lower numbers of passenger exchange with China as compared to other countries. While importation risk index does not directly represent the number of infected passengers imported in a country it is important to have the robust validation of the methodology. For this purpose, a scatter plot between number of confirmed imported cases from various countries and importation risk index is shown in Fig. 3. As evident from the figure a correlation (*R*^2^ = 0.99) clearly indicates the strong agreement between number of passengers imported from a specific country and corresponding importation risk index. There is approximately factor of 6 difference between highest number of passengers (from middle east; ~ 300 passengers) and second highest from United Kingdome (~ 50 passenger). To avoid any bias in the representation of correlation we have also plotted the scatter plot between all the countries (excluding middle east) and importation risk index (show in inset in Fig. 3), and observed the strong relation (*R*^2^ = 0.91).

### Concluding remarks

We used the various online sources along with the data from government organizations for ten countries to screen the flights and passengers form 24 airports connecting various international airports in India. As of 4th March 2020 India has started the thermal screening of passengers arriving from 12 countries, viz. China, South Korea, Japan, Italy, Iran, Singapore, Thailand, Malaysia, Hong Kong, Vietnam, Nepal, and Indonesia. It is, however, not clear if the passengers either travelling and/or transiting through any of the airports form middle east, especially Dubai, were also being subjected to thermal screening. If that was not the case, it is very important to mention that non-screening of passengers arriving through large transit hub like Dubai (DXB) and were infected could have remained undetected, which later on went on to became positive cases. On the other hand, the stamping of the passengers initiated by authorities in India came as one of the most effective steps, as long as it was stringently executed by authorities and followed by passengers for the self-quarantined for the prescribed period. We strongly emphasize that this was one of the most effective way to contain the spread due to importation of the infected passengers in two following ways. a. a full database of the passenger was being obtained at the time of the screening, and b. it is much easier and feasible to track the very same passenger for next 14 days, which is the advisable quarantine period. Such a screening and additional measures like stamping need not be restricted for the passengers arriving only from largely affected countries but must be extended to the passengers coming from large transit hub in future. For example, at the time of initiating the thermal screening for the passengers arriving from Singapore, there were not many cases registered in Singapore or other east Asian countries. As per our understanding such a measure was implemented primarily to identify the passengers arriving from China, which was epicenter of pandemic, transiting through one of the east Asian countries particularly Singapore airport. Overall, we believe that the effective measures taken by the government and authorities at various international airports in India were adequate and effective, although they were restricted only to the passengers arriving from largely infected countries. We, however, also note that the screening and quarantining of the passengers arriving from major transit hub could also have been strictly implemented from the very beginning. We also recommend that international flight operations in India could be cautiously opened in phased manner. We are also of the opinion, based on our analysis, that only one airport can be considered for international operations until the global pandemic is substantially brought under control. Neverthless, these policies are just suggestions and can be reviewed periodically.  

It is very important to note that analyses presented here involve the modeling and estimation based on combination of various different data sources. Under such a situation, where large datasets from various sources is combined to derive certain index, requires a lot of assumptions to consider, and pose various limitations. All these simulations and subsequent estimation heavily rely on the accuracy and reasonable assumption of passenger seats and flight data. In this way, we are not aware of any particular methodology to validate our model, except that comparing the derived importation risk index with actual number of passengers, which for our study is in good agreement. Nevertheless, our results provide data and methodology, which in a nominal way could be used for effective measures and mitigation policies to be implemented at travel hubs for effective containment of travel related risk of infections. Lastly the study presented here completely relies on the data available at this period of time and can be further tuned in future as and when additional data is available, which may substantially alter the results/conclusions presented here.

## Data Availability

The data, codes, and post processed data used in this this study are available from the corresponding author upon reasonable request.
